# Ultrasound-Induced *Escherichia coli* O157:H7 Cell Death Exhibits Physical Disruption and Biochemical Apoptosis

**DOI:** 10.3389/fmicb.2018.02486

**Published:** 2018-10-16

**Authors:** Jiao Li, Luyao Ma, Xinyu Liao, Donghong Liu, Xiaonan Lu, Shiguo Chen, Xingqian Ye, Tian Ding

**Affiliations:** ^1^Department of Food Science and Nutrition, National Engineering Laboratory of Intelligent Food Technology and Equipment, Zhejiang University, Hangzhou, China; ^2^Key Laboratory for Agro-Products Postharvest Handling of Ministry of Agriculture, Zhejiang Key Laboratory for Agro-Food Processing, Hangzhou, China; ^3^Food, Nutrition and Health Program, Faculty of Land and Food Systems, The University of British Columbia, Vancouver, BC, Canada

**Keywords:** ultrasound, sublethal effect, irreversible lethal effect, physical disruption, bacterial apoptosis

## Abstract

Ultrasound has attracted great interest of both industry and scientific communities for its potential use as a physical processing and preservation tool. In this study, *Escherichia coli* O157:H7 was selected as the model microbe to investigate the ultrasound-induced cell death. Slight variations in membrane potential and ion exchanges across membrane induced by low-intensity ultrasound increased the membrane permeability of *E. coli* O157:H7, and this reversible sublethal effect can preserve the viability of *E. coli* O157:H7 and meanwhile be beneficial for bioprocessing application. In comparison, high-intensity ultrasound resulted in irreversible lethal effect on *E. coli* O157:H7, which can be applied in the field of microbial inactivation. In addition, both low- and high-intensity ultrasound induced either physical destruction or trigger genetically encoded apoptosis of *E. coli* O157:H7. Accumulation of reactive oxygen species and decrease of adenosine tri-phosphate might be related to the physiological and biochemical hallmarks of apoptosis, including exposed phosphatidylserine and activated caspases in *E. coli* O157:H7. The result provides novel insight into the mechanisms of non-thermal physical treatment on the inactivation of bacteria and lays foundation for the further research on the cell signaling and metabolic pathway in apoptotic bacteria.

## Introduction

Ultrasound is a type of vibrational energy generated by sound waves with frequency above the audible limit of human hearing. It can be divided into two major categories, namely high-frequency ultrasound (2–20 MHz) and power ultrasound (20–100 kHz) (Feng et al., 2008). The former is typically used for medical imaging and non-destructive inspection (Kvame and Vangen, 2007; Ninoles et al., 2010), while the latter has been widely applied in diverse areas, such as extraction of bioactive components and inactivation of microbes and enzymes (Ercan and Soysal, 2011; Gao et al., 2014b). Power ultrasound waves can create a series of expansion and compression cycles when cavitation bubbles pass through the media, referred as the acoustic cavitation (Kentish and Feng, 2014; Pinela and Ferreira, 2017). In the stable cavitation cycle, the collapse of bubbles does not occur but can still produce micro-streaming and extensive shear stresses. In the transient cavitation cycle, the collapse of bubbles creates various physical and chemical effects, such as localization of hot spots, creation of liquid jets and shock waves, as well as production of free radicals and hydrogen peroxide (Ashokkumar, 2011; Chemat et al., 2011; Jose et al., 2014). For the power ultrasound, the low-intensity ultrasound is applied to damage cell walls and liberate intracellular components in the field of bioprocessing. It provides an efficient approach to increase membrane permeability while induces no severe damages on the intracellular structures. In comparison, the high-intensity ultrasound can lead to the physical disruption of bacterial structures, including cell wall, cytoplasmic membrane, and intracellular structures. Thus, it is regarded as an irreversible nonthermal inactivation technique (Li et al., 2016) and has been widely applied in wastewater treatment and food processing due to its enhanced penetration to the inaccessible sites, less energy cost, greater homogeneity, and less effect on food quality and nutritional contents compared to the conventional thermal processing techniques (Alvarez et al., 2003; Piyasena et al., 2003; Bilek and Turantas, 2013; Arvanitoyannis et al., 2017).

Although the inactivation efficacy of ultrasound treatment on microorganisms has been comprehensively studied, the potential influence on molecular mechanisms of the microbes is still in its infancy. For instance, besides the physical disruption of bacterial cell walls, ultrasound treatment demonstrates the ability to induce mammalian cell apoptosis (Ashush et al., 2000; Feril et al., 2005). Apoptosis is defined as genetically encoded programmed cell death, associating with appropriate development, and homeostasis in the multicellular organisms (Ramsdale, 2012). It typically involves the deployment of a family of cysteine-dependent regulatory proteases with aspartate specificity (Strasser et al., 2000). At the cellular level, it is exhibited as the exposure of phosphatidylserine on the outer membrane leaflet, chromatin condensation, nuclear fragmentation, and loss of mitochondrial membrane potential (Arambage et al., 2009). Recent studies have identified that apoptosis also conserves in single-cell organisms like bacteria. Typical biochemical hallmarks of apoptosis were observed in microorganisms when response to a large variety of intra- and extra-cellular stimuli and stresses (Erental et al., 2012; Ramsdale, 2012). For example, Lunov et al. (2016) discovered that a short-term plasma exposure could induce the accumulation of reactive oxygen species (ROS), resulting in apoptosis of *Escherichia coli*, *Pseudomonas aeruginosa*, and *Staphylococcus aureus*. Typical apoptosis hallmarks were also identified in *E. coli* O157:H7 dead cells after antibiotic treatment (Dwyer et al., 2012), termed as apoptosis-like death (Tanouchi et al., 2013). Hence, it is of great importance to study whether the ultrasound treatment can induce apoptosis-like death under various ultrasound conditions (e.g., intensity and duration).

In the current study, *E. coli* O157:H7 was selected as the model microbe to investigate the ultrasound-induced cell death. The sublethal and lethal effects of different ultrasonic intensities on *E. coli* O157:H7 were compared and its impact on bioprocess/inactivation applications was discussed. The ultrasound-induced membrane permeability was also investigated, including the variations in membrane potential and ion channels. Besides the physical disruption, bacterial apoptosis during ultrasound treatment was illustrated for the first time. The hallmarks of apoptosis and potential apoptosis-inducing factors were studied as well.

## Materials and Methods

### Bacterial Strains and Preparation of Bacterial Cell Suspensions

*Escherichia coli* O157:H7 NCTC 12900 was purchased from China Center of Industrial Culture Collection. The stock culture was streaked on eosin-methylene blue agar plate and then a single colony was transferred into 100 mL of nutrient broth (Hope BioTech Co. Ltd, Qingdao, Shandong, China) and incubated at 37°C for 24 h to reach the stationary phase (∼10^9^ CFU/mL). Cells were harvested by centrifugation at 2,320 × *g* for 10 min at 4°C and washed twice using 0.85% (w/v) sterile saline solution.

### Ultrasound Treatment

A 10-mm-diameter ultrasonic processor probe (JY92-IIDN, Scientz, Ningbo, Zhejiang, China) was used as the stationary treatment system in this study. Thirty milliliters of the diluted bacterial cell suspension (10^8^CFU/mL) was added into a cylindrical tube (85 mL), followed by positioning the ultrasonic probe 2 cm below the surface of the bacteria suspension. Power density (*D*, W/mL) of ultrasound dissipated into the medium with volume *V* is given by *D* = *P*/*V*, where *P* is the input power. Power intensity (*I*, W/cm^2^) dissipated from a probe tip with radius r is given by *I* = *P*/(π*r*^2^). The sonication was performed using defined ultrasonic frequency (20 kHz), power density (0.667 and 6.67 W/mL), power intensities (25.5 and 255 W/cm^2^), and duration times (0, 5, 15, 25 min). In order to avoid the potential thermal effect, the ultrasound system was equipment with a circulating thermostatic bath (DC-1006, Safe Corporation, Ningbo, Zhejiang, China) that can control the heating and refrigerating. The sample temperature was measured with a thermometer located in the center of the glass tube, which was controlled at about 25 ± 1°C.

### Antimicrobial Effect of Ultrasound

The survival of *E. coli* O157:H7 cells was evaluated after ultrasonic treatment using the conventional plating assay (Davey, 2011). Briefly, untreated and ultrasound-treated bacterial suspensions were 10-fold serially diluted in 0.85% (w/v) sterile saline solution. One milliliter of the diluted mixture was plated onto non-selective tryptic soy agar (TSA) (Hope Bio-Technology Co., Ltd., Qingdao, Shandong, China), as well as selective medium TSA supplemented with 2% (w/w) sodium chloride. Plates were incubated at 37°C for 24 h in the aerobic condition. Due to the alterations in membrane permeability, sublethal injured bacteria were not able to recover and grow on the selective media. Therefore, sublethal rate (%) is calculated according to the equation of (1-*N*_selective_/ *N*_non-selective_) ×100, where *N*_selective_ (CFU/mL) and *N*_non-selective_ are the numbers of *E. coli* O157:H7 grown on the selective and non-selective medium, respectively.

### Membrane Potential Measurement

The BacLight^TM^ Bacterial Membrane Potential Kit (Invitrogen, Grand Island, NY, United States) was used to measure cell membrane potential. According to the manufacturer’s guideline, 10 μL of 3 mM 3,3′-diethyloxacarbocyanine iodide [DiOC_2_(3)] was added into 1 mL of bacterial sample and mixed thoroughly. As for the depolarized control, 10 μL of 500 μM carbonyl cyanide 3-chlorophenylhydrazone (CCCP) was mixed with 1 mL of bacterial sample before the addition of DiOC_2_(3). The mixture was incubated at 25°C for 30 min, followed by analysis in a flow cytometer (Beckman Coulter Inc., Miami, FL, United States).

### Quantification of Extracellular Potassium Ion (K^+^)

Determination of potassium in the bacterial cell-free supernatant was carried out using the/a flame atomic absorption spectrometry (AA 7000, Shimadzu, Kyoto, Japan). The analysis was performed using a hollow cathode lamp at 766.5 nm with a current of 10 mA and a slit width of 0.5 nm. An acetylene-air flame was used. The gas flow rate and the burner height were adjusted to 2.0 L/min and 7 mm, respectively.

### Quantification of Intracellular Calcium Ion (Ca^2+^)

The amount of free cytosolic calcium in bacterial samples was determined using cell-permeable probe Fluo-3 AM (Beyotime, Shanghai, China). Samples were incubated with 5 μM Fluo-3 AM at 37°C for 60 min. After staining, bacterial cells were washed with sterile saline solution and incubated for another 20 min to ensure Fluo-3 AM could be fully converted to Fluo-3 by intracellular esterase. The Fluo-3 was able to combine with Ca^2+^ and show high fluorescence intensity, which was determined using a fluorescent microplate reader (BioTek Instruments, Inc., Winooski, VT, United States) at excitation and emission wavelengths of 485 and 525 nm, respectively.

### Scanning Electron Microscopy

The control and treated bacterial samples were centrifuged at 8,000 × *g* for 10 min to collect bacterial pellets and washed twice with 0.85% (w/v) sterile saline solution. The samples were then fixed with 2.5% (v/v) glutaraldehyde (TAAB) for over 4 h and washed three times with phosphate buffer (0.1 M; pH 7.0). The cells were post-fixed with 1% (w/v) osmium tetroxide for 1–2 h and washed three times with phosphate buffer (0.1 M; pH 7.0). The samples were then dehydrated using a series of ethanol aqueous solution (30, 50, 70, 80, 90, 95, and 100%), transferred to a mixture of ethanol and iso-amyl acetate (v:v = 1:1) for ∼30 min, and then placed in pure iso-amyl acetate and incubated overnight. The dehydrated samples were coated with gold–palladium and characterized using a Hitachi Model SU8010 Scanning Electron Microscopy (SEM) (Tokyo, Japan).

### Phosphatidylserine Exposure Analysis

The externalization of phosphatidylserine in the apoptotic cells was assessed using FITC Annexin V/Dead Cell Apoptosis Kit (Invitrogen, Grand Island, NY, United States). First, 5 μL of FITC-labeled annexin V and 1 μL of propidium iodide (PI; 100 μg/mL) were added to 100 μL of bacterial cell suspension. After incubation at 25°C for 15 min, cell analysis was performed using a Gallios flow cytometer equipped with a 488-nm argon-ion laser for excitation. A gate named “[A]” created in the dot plot of forward scatter versus side scatter was preset to distinguish bacteria from artifacts. The Kaluza software package (Beckman Coulter Inc., Miami, FL, United States) was used to analyze the flow cytometry data.

### Determination of Caspase Activity

The CaspGLOW^TM^ Fluorescein Active Staining Kit (Invitrogen, Grand Island, NY, United States) was used to determine the active caspases in *E. coli* O157:H7 cells. After sonication, bacterial cells were incubated with FITC-conjugated, pan-caspase inhibitor peptide Z-VAD-FMK for 60 min, followed by cell analysis using flow cytometry as aforementioned.

### Quantification of Extracellular Hydrogen Peroxide (H_2_O_2_)

The concentration of H_2_O_2_ generated in the medium was determined using a Hydrogen Peroxide Assay Kit (Beyotime, Shanghai, China) following the manufacturer’s guidelines. After ultrasound treatment, 50 μL of bacterial sample was placed into an ultraviolet transparent 96-well plate (Corning Inc., New York, United States), and then 100 μL of the detection reagent was added and incubated at 25°C for 30 min. The absorbance at 560 nm was measured using a spectrophotometer (Thermo Scientific Inc., Waltham, MA, United States).

### Determination of Intracellular Reactive Oxygen Species

The levels of ROS were determined using a cellular assay probe 2,7-dichlorofluorescin diactate (DCFH-DA; Beyotime, Shanghai, China). Before ultrasound treatment, bacterial cells were incubated with DCFH-DA at a final concentration of 10 μM at 37°C for 20 min. Then, 200 μl of the sample was transferred into a 96-well black bottom plate (Fisher Scientific, Loughborough, Leicestershire, England) and measured using a fluorescent microplate reader at the excitation and emission wavelengths of 485 and 525 nm, respectively.

### Measurement of Adenosine Tri-Phosphate (ATP)

Intracellular ATP levels were determined using a Bac Tiler-Glo Microbial Viability Assay Kit (Promega, Madison, WI, United States). After ultrasound treatment, 100 μL of the sample solution was added into an opaque 96-well plate (JingAn Biological Technology Co., Ltd, Shanghai, China), followed by the addition of 100 μL of BacTiter-Glo^TM^ Regent. The mixture was incubated for 5 min. A multimode microplate reader with luminescence measurement ability (Berthold Technologies GmbH & Co. KG, Bad Wildbad, Germany) was used to measure the luminescence intensity.

### Statistical Analysis

All of the experiments were repeated at least for three times. The data were analyzed by ANOVA using the SPSS Statistics 20 software (IBM Co., Armonk, NY, United States) and expressed as mean ± standard deviation. A *P*-value of less than 0.05 indicated a significant difference between different treatment groups.

## Results and Discussion

### Antimicrobial Effect of Low- and High-Intensity Ultrasound

The survival of *E. coli* O157:H7 treated with low- and high-intensity ultrasound for different time periods is summarized in **Table 1**. Bacterial cell counts were significantly different (*P* < 0.05) between selective and non-selective media assays during low-intensity ultrasonic processing. The sublethal rates of *E. coli* O157:H7 were 30.26, 21.59, and 17.05% after 5, 15, and 25-min exposure, respectively. This result may be attributed to the reversible membrane permeabilization induced by low-intensity ultrasonic treatment (Pitt and Ross, 2003), such as temporary “opening” pores on the membrane lipid bilayers (Yeo and Liong, 2013). In contrast, there was no significant difference (*P* > 0.05) in bacterial cell counts between selective and non-selective media assays during high-intensity ultrasonic processing, indicating that the amount of sublethally injured *E. coli* O157:H7 cells was negligible. This result was in agreement with our previous studies that high-intensity ultrasound was an “all or nothing” process (Li et al., 2017a,b). Lethal and sublethal effects of ultrasound treatments on *E. coli* O157:H7 cells depend upon both intensity and duration. The sublethal effects caused by low-intensity ultrasound can accelerate the transportation of intercellular macromolecules across cell membranes and at the same time preserve bacterial viability, which is beneficial for bioprocess application (Yeo and Liong, 2013). However, sublethal effects should be eliminated for efficient microbial inactivation, because pathogenic bacteria can repair the damage of cytoplasmic membrane under the favorable growth condition, posing significant threats to food safety and public health (Guillier et al., 2005). Taken together, an appropriate selection of intensity as well as duration of ultrasound treatment is critical to different applications. When applied in the field of bioprocessing, low-intensity, and short-duration should be selected for reversible changes of cytoplasmic membrane. In comparison, high-intensity and long-duration can be applied in microbial inactivation for its irreversible lethal effect on bacteria.

**Table 1:** Viable and sublethal *E. coli* O157:H7 populations after low- and high-intensity ultrasound treatments.

Time (min)	Low-intensity ultrasound		High-intensity ultrasound	
	TSA (log CFU/mL)	TSA + 2%NaCl (log CFU/mL)	TSA (log CFU/mL)	TSA + 2%NaCl (log CFU/mL)
5	7.91 ± 0.08^c,C^#	7.63 ± 0.07^b,B^	7.13 ± 0.07^c,A^	7.07 ± 0.05^c,A^
15	7.49 ± 0.06^b,C^	7.31 ± 0.07^a,B^	6.89 ± 0.09^b,A^	6.86 ± 0.10^b,A^
25	7.02 ± 0.05^a,C^	6.89 ± 0.06^a,B^	6.13 ± 0.07^a,A^	6.09 ± 0.06^a,A^

*Initial bacterial population was approximately 8.27 ± 0.04 log CFU/mL. ^#^Values are the mean of triplicate measurements ± standard deviation; values with different lowercase letters in the same column and uppercase letters in the same row showed a significant difference at P < 0.05.*

### Changes of Membrane Permeability

**FIGURE 1:**
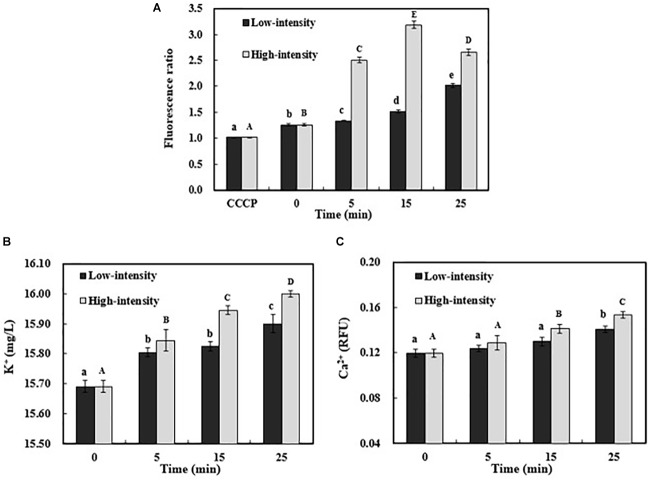
Membrane potential changes of *E. coli* O157:H7 after ultrasound treatments were expressed as the ratio of red and green fluorescence intensity **(A)**. The extracellular K^+^ **(B)** and intracellular Ca^2+^ concentration **(C)** of *E. coli* O157:H7 during ultrasound exposure. Data were expressed as means ± standard deviation (*n* = 3). Different lowercase letters demonstrated a significant difference at *P* < *0.05*.

The membrane permeability is an important indicator of the final fate of *E. coli* O157:H7. In this case, the potential ultrasound-induced membrane permeability was further investigated, including ion channels and membrane potential. Various ions have an asymmetric distribution across the membrane (Anand et al., 2017). Potassium ion (K^+^) has an increasing trend from outside to inside of the cell membrane, while sodium (Na^+^) and chloride (Cl^-^) ions have the opposite distribution pattern. These ionic concentration gradients across cell membrane provide the potential energy to drive the formation of membrane potential (Wadekar and Vidic, 2017). Membrane potential plays an important role in cell physiological processes, especially energy generation and substance transportation (Jiao et al., 2004; Lunde et al., 2009). As shown in **Figure 1A**, the positive control was treated by CCCP, resulting in totally depolarized membrane potential. As the treatment time increased, the hyperpolarized membrane potential of *E. coli* O157:H7 gradually occurred in low-intensity ultrasonic group. In contrast, the membrane potential increased dramatically and reached to the maximum value at 15 min when treated with high-intensity ultrasound. The hyperpolarization of membrane might be related to the opening of ion channels on the cell membrane (Sokabe et al., 2015). During ultrasonic processing, the efflux of intracellular K^+^ via the ion channel elevated the extracellular K^+^ concentration (**Figure 1B**). In addition, the extracellular K^+^ concentration after high-intensity sonication was significantly higher (*P* < 0.05) than other treatment groups, which might be related to the breakdown of cell envelopes and the leakage of cellular contents. In response to the changes in membrane potential, the voltage-dependent calcium channel could also be activated, resulting in the influx of Ca^2+^ in *E. coli* O157:H7 cells for a longer duration treatment (**Figure 1C**). This might explain that the cell membrane potential started to decrease after treatment by high-intensity ultrasound for 25 min (**Figure 1A**). Ca^2+^ plays a critical role in signal transduction pathways and acts as a second messenger in cells. The excessive entry of Ca^2+^ into the cells may cause cell damage or even lead to cell apoptosis (Berridge, 2017; Lazniewska and Weiss, 2017). Overall, the alteration of membrane potential and ion exchanges across membrane could increase the membrane permeability and change the cell function.

### Physical Destruction Induced by Ultrasound

**FIGURE 2:**
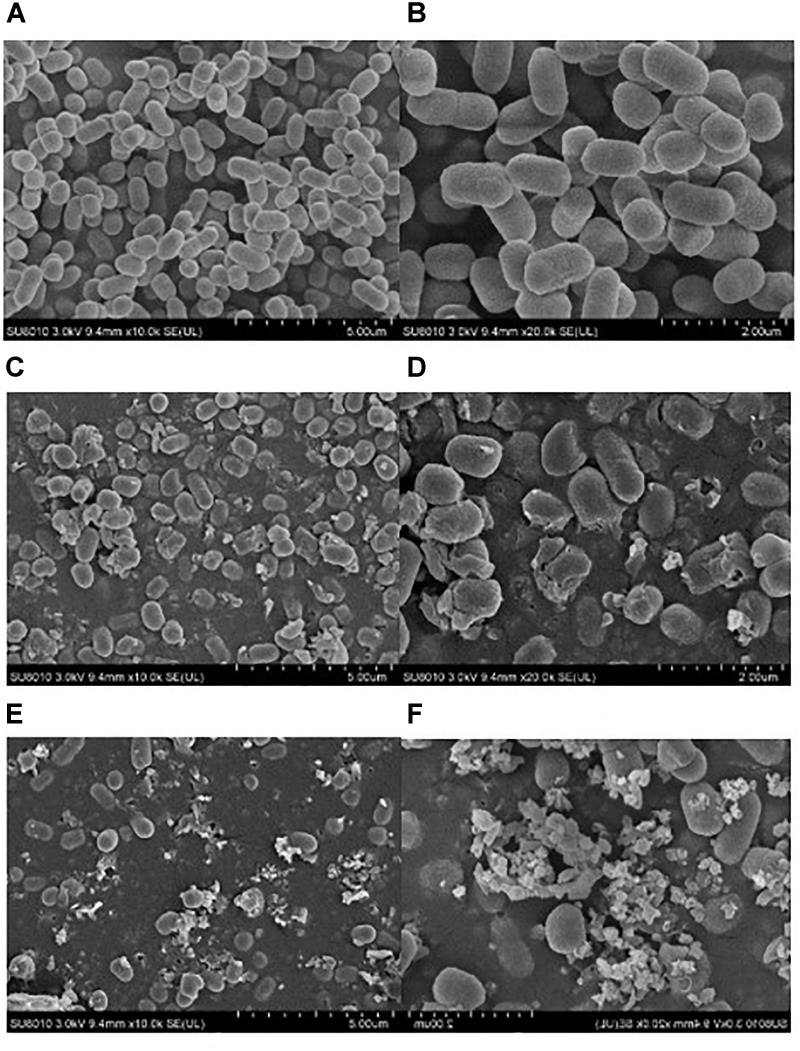
SEM images of *E. coli* O157:H7 cells: untreated bacteria **(A,B)**, bacteria treated with low-intensity ultrasound for 25 min **(C,D)**, and bacteria treated with high-intensity ultrasound for 25 min **(E,F)**.

Physical effects of ultrasound have been recognized as the major action in bacterial inactivation (Ashokkumar, 2011; Chemat et al., 2011). In this study, SEM analysis was conducted to reveal the morphological damage of *E. coli* O157:H7 cells after the ultrasound treatment. **Figure 2** indicated that the level of destruction of *E. coli* O157:H7 cells was dependent upon the ultrasound intensity. The untreated *E. coli* O157:H7 cells were in the rod shape with smooth cell walls and cell membranes (**Figures 2A,B**). After sonication treatment at low intensity for 25 min, most *E. coli* O157:H7 cells maintained the original shape, but part of the cells was physically damaged into fragments (**Figure 2C**). In **Figure 2D**, rough and blurry cell envelopes were observed although the cell shape of *E. coli* O157:H7 did not change. Furthermore, some *E. coli* O157:H7 cells showed pore formation and localized rupture after treatment (**Figure 2D**). These results altogether further explained the existence of sublethal effects after low-intensity ultrasound treatment. Compared to the low-intensity ultrasound treatment, high-intensity ultrasound treatment produced more severe physical damage on *E. coli* O157:H7 cells (**Figures 2E,F**). Notably, *E. coli* O157:H7 cells were either completely damaged into debris or survived with an intact morphological profile after the high-intensity ultrasound was applied. Therefore, the observation supported our assumptions that high-intensity ultrasound causes “all or nothing” effect on the bacterial cells.

### Biochemical Apoptosis Induced by Ultrasound

**FIGURE 3:**
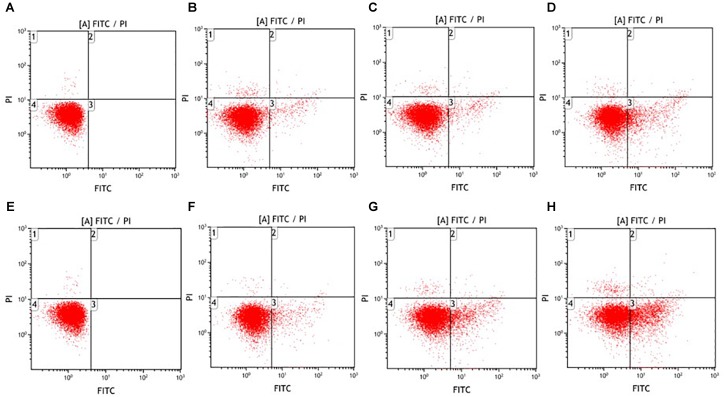
Fluorescence dot plots of *E. coli* O157:H7 in response to staining with FITC-labeled annexin-V and PI after low- **(A–D)** and high-intensity **(E–H)** ultrasound treatments. Duration of treatment was 0 min **(A,E)**, 5 min **(B,F)**, 15 min **(C,G)**, and 25 min **(D,H)**.

Besides the investigation on the physical disruption of *E. coli* O157:H7, the biochemical hallmarks of cell apoptosis were also characterized after exposure to the ultrasound treatment. In the apoptotic cells, phosphatidylserine translocates from the inner to the outer leaflet of the cytoplasmic membrane (Chen et al., 2013; Nagata et al., 2016). By using flow cytometry, phosphatidylserine exposure could be determined through the binding between bacterial cells and two fluorescent reagents. FITC-labeled annexin V is permeable to live and apoptotic cells and propidium iodide (PI) can only stain the dead cells. As shown in **Figure 3**, different subpopulations were identified based upon their differential binding characteristics with FITC-labeled annexin-V and PI (Lunov et al., 2016). Specifically, FITC-positive PI-negative cells (lower right quadrant) exposed phosphatidylserine on cell surface without an increase in membrane integrity, indicating an apoptotic-like death. FITC-positive PI-positive cells (upper right quadrant) were considered to have gone through necrotic-like death. FITC-negative PI-positive cells (upper left quadrant) might be the physically disrupted bacteria. FITC-negative PI-negative cells (lower left quadrant) were related to live cells or cell debris. Both low- and high-intensity ultrasound induced the exposure of phosphatidylserine on the surface of *E. coli* O157:H7 cells. The apoptotic rates were 0.00, 3.15, 5.82, 12.77% for low-intensity treatment and 0.00, 5.62, 13.24, 20.1% for high-intensity treatment after exposure for 0, 5, 15, 25 min, respectively. Notably, the upper left quadrant showed ultrasound-induced bacterial death that exhibited a certain proportion of physically disrupted *E. coli* O157:H7 cells. In the meanwhile, almost no necrotic cells were generated, which was considerably different from other common inactivation treatments (Eisenberg et al., 2010; Lunov et al., 2016). For example, it was reported that *E. coli* O157:H7 cells exhibited apoptotic and necrotic scenarios of bacterial death after non-thermal plasma treatment(Lunov et al., 2016). This also applied to hydrogen peroxide (Madeo et al., 1999) and amphotericin B treatment on yeast (Phillips et al., 2003). Therefore, ultrasound either triggered the physical disruption of *E. coli* O157:H7 or exhibited biochemical hallmarks of apoptosis.

**FIGURE 4:**
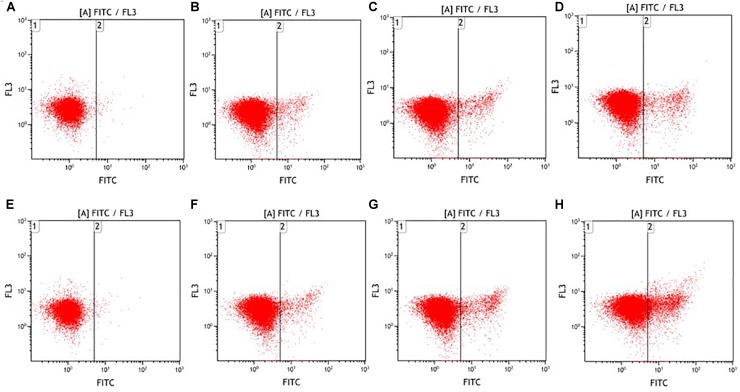
Fluorescence dot plots of *E. coli* O157:H7 in response to staining with FITC-VAD-FMK after low- **(A–D)** and high-intensity **(E–H)** ultrasound treatments. Duration of treatment was 0 min **(A,E)**, 5 min **(B,F)**, 15 min **(C,G)**, and 25 min **(D,H)**.

In addition, we further employed FITC-VAD-FMK to detect cellular caspase activity. The caspases are a family of cysteine proteases with aspartate specificity, which can be activated by pro-apoptotic factors and subsequently lead to the irreversible apoptosis (Los et al., 1999; Liu and Clem, 2011). According to **Figure 4**, ultrasound-induced *E. coli* O157:H7 cell death was associated with the increased caspase activity. The percentages of *E. coli* O157:H7 cells with activated caspases were 0.22, 3.39, 4.70, 8.55% after low-intensity ultrasound and 0.22, 3.91, 7.14, 14.08% after high-intensity ultrasound for 0, 5, 15, 25 min, respectively. The proportion of phosphatidylserine-exposed *E. coli* O157:H7 cells was significantly higher (*P* < 0.05) than that of caspases-activated *E. coli* O157:H7 cells. This might be due to the occurrence of caspase-independent manner during apoptosis, such as catalytically distinct proteases (Broker et al., 2005). Taken together, both low- and high-intensity ultrasound could influence biochemical processes, leading to the programmed cell death in *E. coli* O157:H7 cells.

### Analysis of Apoptosis-Inducing Factors

**FIGURE 5:**
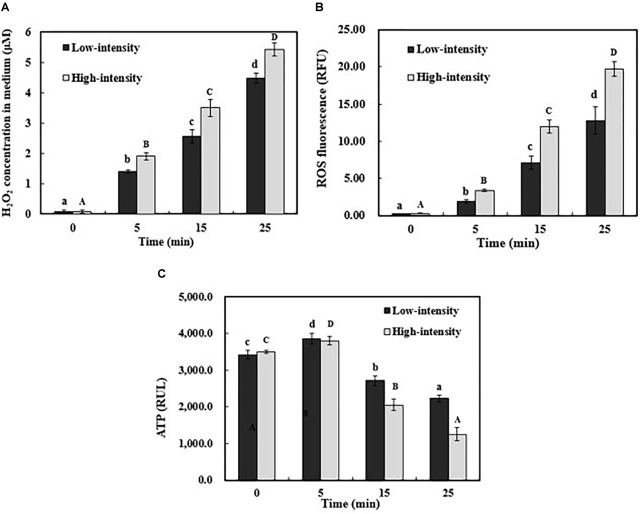
The generation of H_2_O_2_ in medium **(A)**, intracellular ROS **(B)**, and ATP level **(C)** in *E. coli* O157:H7 during ultrasound exposure. Individual results are expressed as means ± standard deviation (*n* = 3). Different lowercase letters showed a significant difference at *P* < 0.05.

To identify the possible apoptosis-inducing triggers, the extracellular H_2_O_2_ concentration was examined, as well as intracellular ROS and ATP level. In this study, the production of H_2_O_2_ increased by 4.40 and 5.33 μM after 25-min low- and high-intensity ultrasound exposure, respectively (**Figure 5A**). The principle of H_2_O_2_ production during ultrasound treatment was previously reported (Sato et al., 2000; Gao et al., 2014a). Hydrogen and hydroxide radicals are produced by the hydrolysis of water inside the cavitation bubbles. Then, subsequent reaction in the interface and the solvent phase may lead to the production of H_2_O_2_, resulting in the variation of oxidative stress in the bacterial cells (Stanley et al., 2004). The intracellular ROS level in *E. coli* O157:H7 was also investigated due to its important role in triggering apoptosis (Burhans et al., 2003; Dwyer et al., 2007; Bayles, 2014). ROS generation can be efficiently eliminated by endogenous oxidation defense system (Tan et al., 1998). Once the level of ROS exceeds the defense capability, the antioxidant defense collapses and results in cell apoptosis (Chandra et al., 2000). As shown in **Figure 5B**, the level of ROS significantly increased by ultrasonic processing (*P* < 0.05). When treated by high-intensity ultrasound for 25 min, the fluorescence intensity of ROS increased from 0.28 to 19.67 RFU (**Figure 5B**). This increase pattern of intracellular ROS level was in agreement with phosphatidylserine exposure and activated caspases. Moreover, the ATP level of *E. coli* O157:H7 cells was examined because the ATP-dependent phosphatidylserine translocation would be significantly affected by the energy level. After 25 min, both low- and high-intensity ultrasound treatments resulted in a remarkable reduction of ATP level (**Figure 5C**). According to the previous studies, flippase can specifically translocate phosphatidylserine between the outer and inner cytoplasmic membrane in an ATP-dependent manner (Balasubramanian and Schroit, 2003). When the ATP level was low, flippase would lose the transport ability and the exposed phosphatidylserine cannot return to the inner cytoplasmic membrane (Seigneuret and Devaux, 1984; Nagata et al., 2016). Our results indicated that the ultrasound exposure could lead to ROS accumulation and ATP reduction, which might result in physiological and biochemical hallmarks of apoptosis in *E. coli* O157:H7. The metabolic pathways induced by these apoptosis-factors need to be investigated in future.

## Conclusion

In this study, it was identified that the lethal and sublethal effects of ultrasound treatments on *E. coli* O157:H7 cells were highly dependent upon ultrasonic intensity and duration. To meet the demands in bioprocess application, low-intensity and short-duration should be selected to increase the membrane permeability and preserve the bacterial viability. In comparison, irreversible lethal effect of high-intensity and long-duration ultrasound exposure can be applied in microbial inactivation. Our study not only demonstrated different physical disruption scenarios induced by ultrasound, but also revealed the relationship between bacterial apoptosis and biochemical processes, such as ROS accumulation and ATP reduction. Further exploration on the metabolic pathways induced by apoptosis-related factors in *E. coli* O157:H7 will be of great significance to elucidate the mechanism of ultrasonic actions.

## Author Contributions

JL and TD drafted the manuscript. All authors listed have edited the manuscript and made substantial and direct contribution to the work. All authors gave approval for publication of the manuscript.

## Conflict of Interest Statement

The authors declare that the research was conducted in the absence of any commercial or financial relationships that could be construed as a potential conflict of interest.
